# Synergistic anticancer effect of CDRI-08 and abiraterone acetate against castration resistant prostate cancer targeting PI3K/Akt pathway

**DOI:** 10.22038/ijbms.2025.85330.18441

**Published:** 2025

**Authors:** Bhavana Jonnalagadda, Sumathy Arockiasamy

**Affiliations:** 1 Department of Biomedical Sciences, Sri Ramachandra Institute of Higher Education and Research, Chennai, Tamil Nadu, India

**Keywords:** Abiraterone, CDRI-08, Combination index, PI3K/Akt pathway, Prostate cancer

## Abstract

**Objective(s):**

There is a considerable interest in combination therapy targeting the complex interlinked pathways in prostate cancer due to the development of drug resistance with monotherapies. A standardized fraction of *Bacopa monnieri *CDRI-08 was developed and patented by the Central Drug Research Institute (CDRI), Lucknow, for the treatment of neurodegenerative diseases. Recent studies with the plant and its phytocompounds have shown effective anticancer and antioxidant activity. Therefore, in the current research, the combined effect of Abiraterone acetate (AA) and CDRI-08 was studied in androgen-independent prostate cancer cells *in vitro*.

**Materials and Methods:**

Initially, the *in vivo* toxicity of CDRI-08 was studied in zebrafish embryos. *In vitro* individual cytotoxicity and the synergistic effect of AA and CDRI-08 were studied in PC3 cell lines with and without growth factors. Nuclear staining with AO/EB and western blotting were performed to analyse apoptotic cell death and changes in protein expression of p-AKT and Casp3 in individual and combination-treated cells.

**Results:**

CDRI-08 has shown no toxicity and teratogenicity in zebrafish embryos. AA and CDRI-08 have shown dose-dependent cytotoxic effects in PC3 cell lines with and without growth factors. Synergism was observed with different concentration ratios of AA and CDRI-08 with and without growth factors, with a good combination index (CI). Apoptosis was observed in individual and combination treated cells with an increase in Casp3 and simultaneous decrease in p-AKT expression levels.

**Conclusion:**

The study confirms the synergistic effect of CDRI-08 and AA at a lower dose, targeting the tyrosine kinase and androgen receptor pathways.

## Introduction

Cancer is one of the leading causes of death worldwide, with prostate cancer being the second leading cause of death in men worldwide (1, 2). The initial treatment methods, including surgery and radiotherapy along with Androgen Deprivation Therapy (ADT)(3), are effective for a shorter duration, as 70% of the patients experience relapse to Castration Resistant Prostate Cancer (CRPC). Studies have shown that the Androgen Receptor (AR) remains an important driver in the recurrence of CRPC, even after the ADT, which is mainly attributed to dysregulated AR pathway and intra-tumoral and adrenal gland androgen synthesis (4). The second-generation drugs, Androgen Receptor Inhibitors (ARIs), function by preventing nuclear localisation and DNA interaction of AR (3). Abiraterone acetate (AA) is an irreversible inhibitor of androgen synthesis enzyme, Cytochrome P450 17A1 (CYP17A1), both in the adrenal glands and CRPC cells. It was the first novel AR pathway inhibitor (ARPI), which has shown effective survival benefits in CRPC patients (5). However, the CRPC cells have shown to develop resistance to AA and proliferate via alternative cellular pathways. 

The analysis of CRPC tissue samples have shown an active AR signaling, along with Epidermal Growth Factor Receptor (EGFR) pathway, Phosphatidylinositol 3-kinase (PI3K)/Akt pathway and the Mitogen Activated Protein Kinases (MAPK) pathway. In normal cells, these signaling pathways are counterbalanced by PTEN, the tumor suppressor gene, which is found to be deleted or mutated in CRPC patients. Thus, in CRPC cells, AR and EGFR/PI3K/Akt/mTOR crosstalk pathway was found to be dysregulated (6, 7), leading to increased cell proliferation and development of drug resistance. The current research strategies target the dysregulated AR, EGFR/PI3K/Akt/mTOR, and other interlinked pathways. As monotherapy in clinical trials, Tyrosine Kinase Inhibitors (TKIs) had shown disappointing outcomes (6). Therefore, a combination therapy targeting complex integrated pathways may increase the activity of the drugs and reduce the chances of the development of drug resistance (8). 

Combination therapy has become a keystone in cancer therapy, which is based on the pharmacodynamics and pharmacokinetics interaction of two or more drugs. It is shown to reduce the chances of drug resistance and increase the activity of the drugs (9). Many pre-clinical and clinical studies of combination therapy with ADT, ARPI, chemotherapy, and TKIs have shown increased proliferation-free survival with reduced effects in CRPC patients (10-12). Phytocompounds with their effective anticancer properties, are also considerable candidates for combination therapy, which have shown to function as chemosensitizers, increasing the effectiveness of conventional chemotherapy drugs (9). 

*Bacopa monnieri*, commonly known as Brahmi, has been in use in Ayurvedic medicine since ancient times for the treatment of neurodegenerative disorders and also as a memory enhancer (13, 14). The Central Drug Research Institute (CDRI) Lucknow has developed a unique formulation of *B. monnieri*, CDRI-08, which was patented (Kahol *et al*., 2003, US6833143). It has shown no systemic pharmacological or toxicological effects at recommended doses (15). The recent research with CDRI-08 was shown to potentiate the germ cell dynamics and reduce peroxidative damage to the sperms, which boosts the spermatogenic process (16). 

The drug-drug interactions in combination studies are categorized as additivity, synergism, or antagonism, which is analyzed with various models (8). The positive interaction, or synergism, occurs when the combined effect of drugs is greater than the expected additive effect. The classical combination model, the Bliss Independence model, is based on the principle of independent activity of drugs at different sites, each contributing to the results outcome. (8, 17). Therefore, in the current study, the cytotoxicity of CDRI-08 targeting the PI3K/AKT pathway was initially evaluated in PC3 cell lines with growth factors (GF). Further, its combination effect with the standard drug, Abiraterone acetate, was also studied 

## Materials and Methods

### Chemicals

CDRI-08 was kindly provided by Lumen Marketing Company, Chennai. Abiraterone acetate was kindly provided by Dr. Sriram Krishnamoorthy, Urologist, SRIHER. Epidermal Growth Factors (EGF) and Dihydrotestosterone (DHT) are the growth factors (GF) used in the study. The primary and secondary antibodies were obtained from Scimax Scientifics, India. All chemicals used in this study were of cell culture grade.

### Cell lines

The androgen-independent human Prostate cancer cell line, PC3 cell lines were obtained from National Centre for Cell Sciences (NCCS), Pune, India. The cells were cultured in DMEM media with 10% FBS, and 1X Antibiotic/Antimycotic and kept at 37 ^°^C, in a humidified atmosphere and 5% CO_2_. The culture medium was changed twice in a week. 

### In vivo toxicity and teratogenic effect of CDRI-08

Maximal Tolerable Concentration (MTC), defines the highest concentration of the sample with no toxicity and teratogenicity. An *in vivo* toxicity study was performed on zebrafish embryos at the same developmental stage for 96 hr post fertilization (hpf). 4 hpf embryos were treated with CDRI-08 (25-400 µg/ml) in E3 medium, positive control (1% Ethanol) and negative control (E3 medium) at 28 ^°^C, and the embryos were studied under a Stereo Zoom microscope (Inverted Nikon SMZ1000). The mortality, hatch rate and teratogenic effects such as, bent body, short body, bent tail. yolk sac edema, and lack of pigmentation, were noted for every 24 hr upto 96hpf. The experiments were repeated thrice and performed in accord with National and Institutional guidelines for the protection of human subjects and animal welfare (18). The 4hpf embryos were treated with CDRI-08 (25-400 µg/ml) in E3 medium. 

### Drug preparation

Stock solutions of AA and CDRI-08 were prepared in DMSO and stored at -20 ^°^C. 10 nM of EGF and DHT were used in the study. Working concentrations of AA (10-30µM) and CDRI-08 (50-250 µg/ml) were freshly prepared in DMEM medium with and without GF. Similarly, the different combination of the AA and CDRI-08 were freshly prepared. 

### In vitro cytotoxicity studies

***MTT assay***

***Individual effect***

The 70% confluent PC3 cells were trypsinized and seeded into a 96-well plate at a density of 5×10^3^ per well. After 24 hr, the cells were individually incubated with different AA and CDRI-08 concentrations. After 48 hr, the cells were rinsed with PBS and incubated with 100µL of MTT for 4 hr. The MTT metabolites were dissolved in 100µL DMSO and incubated at 37 ^°^C for 30 min (19). The absorbance was read at 540nm, with a reference wavelength of 630nm, using a Multiskan Ascent microplate reader (Thermo Labsystems, Franklin, MA, USA). 

The dose-response effects of CDRI-08 and AA were studied, and inhibitory concentrations (IC), IC50, IC25, and IC10 were calculated. Further, the PC3 cells were treated with different ICs of AA and CDRI-08 in the presence and absence of GF, and an MTT assay was performed to validate the IC values.

### Combination effect

The combined effect of different IC ratios (IC10, IC25, and IC50) of CDRI-08 and AA was studied with an MTT assay in the presence and absence of GF (20). 

### Combination index evaluation

The combination effect was analysed with the combination index (CI) (8). The assessment is based on evaluating the cytotoxic effect of individual and combination ICs. The observed combination effect is expressed as a probability, 0≤EAB≤1, and compared with the expected effect by the common formula for probabilistic independence

$\mathrm{CI}=\frac{\mathrm{EA}+\mathrm{EB}+\mathrm{EAEB}}{\mathrm{EAB}}$

Where EA and EB are the observed inhibition rates with CDRI-08 at dose *a* and AA at dose b and EAB is observed combination effect (0≤EA/EB/EAB≤1). 

### Apoptosis analysis

Apoptosis-induced morphological changes in PC3 cell-treated individuals and combination ICs of CDRI-08 and AA were studied with Acridine Orange and Ethidium Bromide (AO/EB) dual staining. Cells were cultured in 24 well plates at the density 1x10^6^ cells in DMEM media with 10% FBS. After 24 hr, the cells were treated with individual and combination ICs of CDRI-08 and AA. After 48 hr, the cells were stained with AO/EB, and the apoptosis-induced effect was characterized by chromatin condensation and yellow and orange staining (21).

### Western blot analysis

PC3 cells were plated into 6 dishes (6×10^5^ cells/dish) with DMEM media with 10% FBS, and after 24 hr, the PC3 cells were treated with individual and combination ICs for 48 hr. Then, the cells were lysed on ice, and the cell lysate obtained was centrifuged at 10,000 rpm at 4 ^°^C for 15 min to remove the cell debris. The protein concentration was estimated in the supernatant (22). The protein samples (30 μg) were separated using 12% SDS-PAGE gels and transferred to nitrocellulose membrane. The membrane was incubated overnight with primary antibodies against GAPDH, ERK, p-ERK, Akt, p-Akt, and Casp3 at 4 ^°^C and then incubated with horseradish peroxidase-conjugated secondary anti-mouse IgG antibody for 1 hr at room temperature. Gel doc (Diversity 4, Syngene, India) was used to visualize the chemiluminescence (23). 

### Data analysis and statistics

All the experiments were performed as three individual experiments in triplicates. The values were expressed as mean±standard deviation (SD). The Student’s t-test and the one-way ANOVA test were used to compare differences in the mean values, and a *P*<0.05 was considered statistically significant. The data were processed in GraphPad Prism 5 (GraphPad Software) and the Synergyfinder tool (24). 

## Results

### In vivo toxicity effect of CDRI-08 in zebrafish embryos

**Figure 1:**
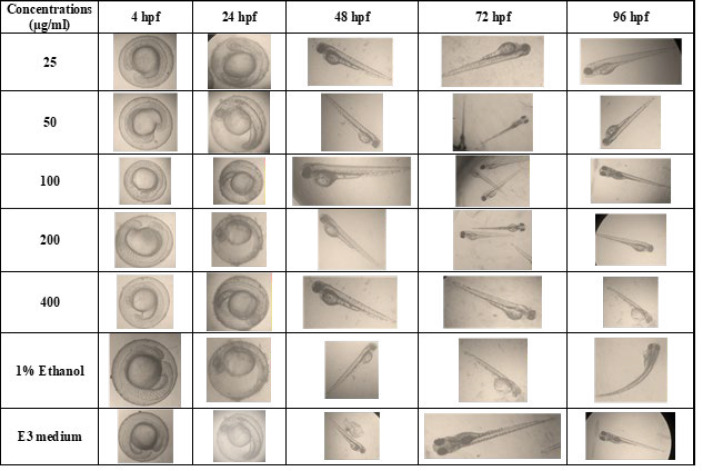
Dose-dependent teratogenic effect of CDRI-08 on zebrafish embryos

In the present study, at 96hpf, the CDRI-08 treated embryos (25, 50, 100 and 200 µg/ml) showed 100% viability (*P*<0.05), with a good hatch rate and no teratogenicity (Figure 1). However, in 400 µg/ml CDRI-08, the viability rate was reduced to 70%, and teratogenicity was observed in 20% (*P*<0.05) of the embryos. The toxicity of CDRI-08 was evaluated using 1% ethanol as the positive control (60% viability) and E3 medium as the negative control (100% viability). 

### In vitro cytotoxicity effect

*Individual effect of AA and CDRI-08*

**Figure 2:**
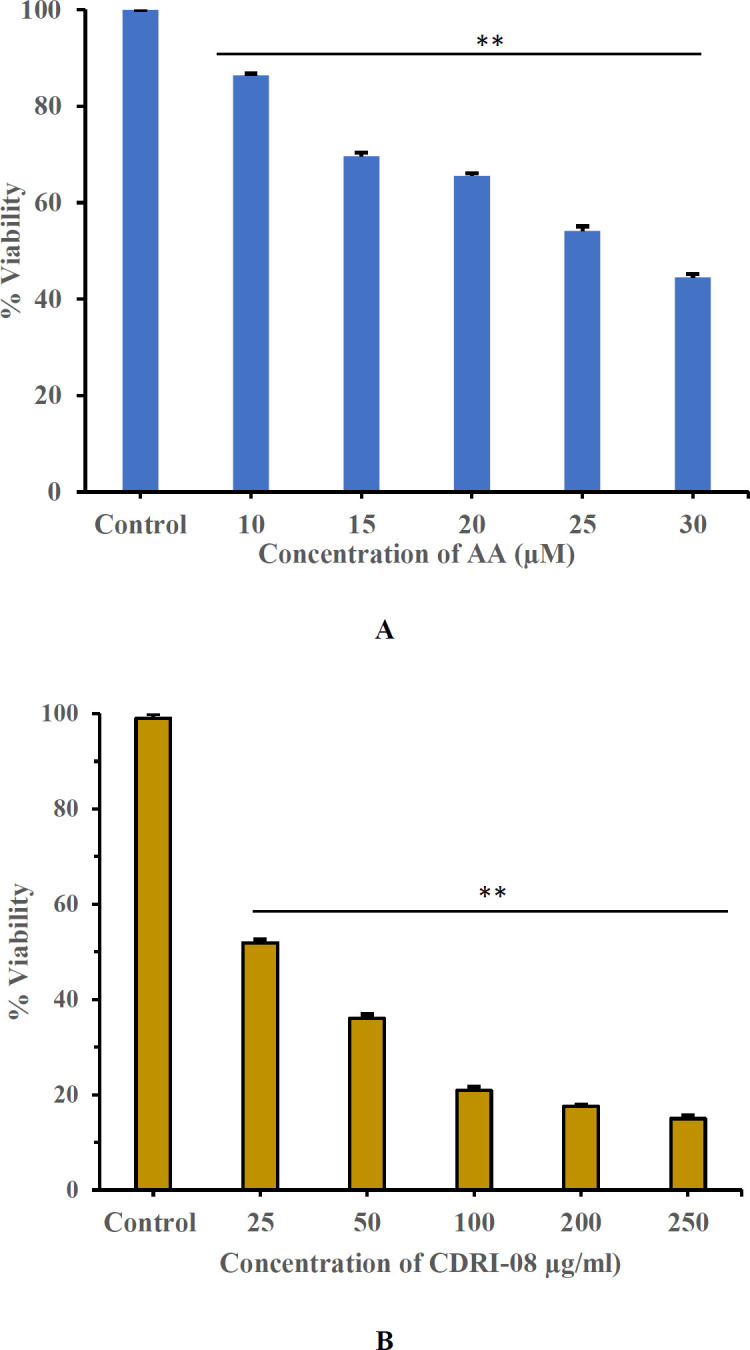
Dose-dependent individual cytotoxic effect of (A) Abiraterone acetate (AA) and (B) CDRI-08 on PC3 cell lines for 48 hr

The standard drug, AA, has shown a dose-dependent cytotoxic effect in PC3 cell lines with a significant decrease in cell viability to 45% at 30 µM (*P*<0.05)(Figure 2A) and an IC50 value of 27 µM. 

The CDRI-08 has also shown a dose-dependent cytotoxic effect in PC3 cell lines, and the cell viability was reduced to 12% at 250 µg/ml (*P*<0.05) (Figure 2B). The IC50 value was found to be 33.6 µg/ml, and 34 µg/ml of CDRI-08 was used further in the combination study. 

Concerning the IC50 values, the corresponding IC25 and IC10 values were also validated both in the presence and absence of GF, as shown in Table 1. 

### Combination effect with AA and CDRI-08

With the estimated cytotoxic effect of AA and CDRI-08 individually, the drug synergy (∼70%) was analysed with the Bliss Independent model using the software SynergyFinder. 

**Figure 3:**
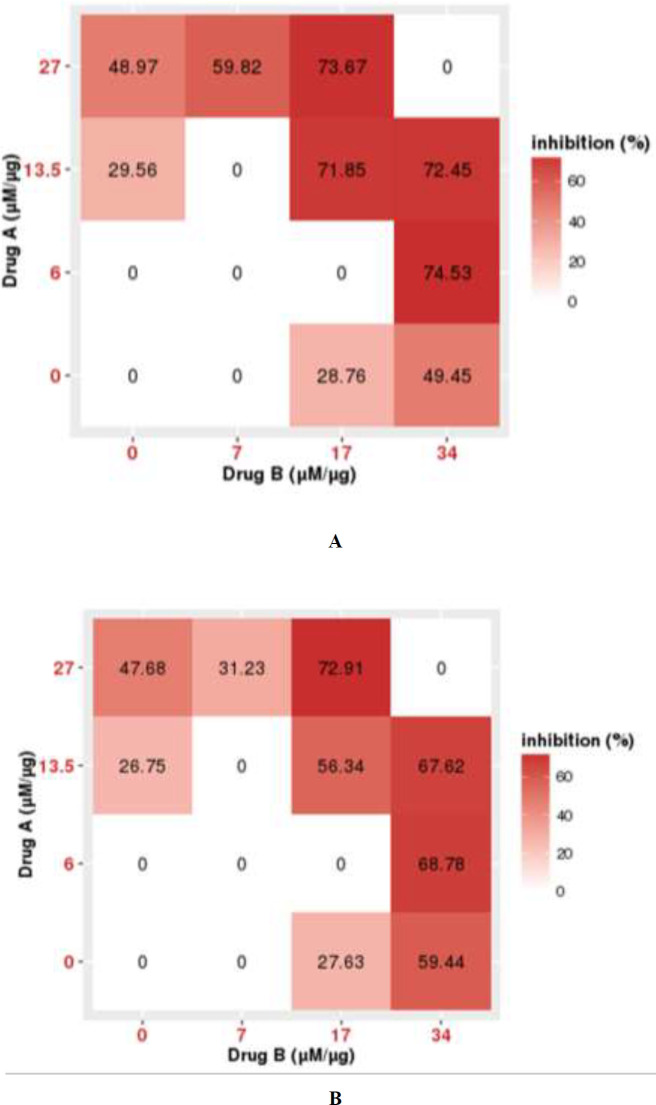
Dose-response matrix of combination effect of Abiraterone acetate (AA) and CDRI-08 in the (A) absence of GF and (B) presence of growth factor (GF)

The combination ICs of AA and CDRI-08 showed significant synergistic cytotoxic effects in PC3 cells, with and without GF, compared to the individual ICs. The C1 with IC25 of AA and CDRI-08 has demonstrated more than a 50% (*P*<0.05) decrease in cell viability with and without GF. The C2 and C5 combination has also shown more than 70% (*P*<0.05) cytotoxic effect. The cytotoxic effect of combination ICs of AA and CDRI-08 in PC3 cells is represented as the dose-response matrix in Figure 3A and B.

Following this, the combination index (CI) was calculated (Table 2), which was found to be less than 1 for C1, C2, C4, and C5, indicating synergism. The CI for C3 was found to be more than 1, representing antagonism. The CI for C1 and C4 in the absence of GF was found to be the lowest, 0.61 and 0.7 (*P*<0.05), respectively, indicating an effective synergism, while the same in the presence of GF was found to be 0.78 and 0.8 (*P*<0.05). The CI values confirm an enhanced cytotoxic effect of AA with CDRI-08.

### AO/EB staining

The apoptotic cell death was studied in PC3 cells treated with individual and combination ICs of CDRI-08 and AA with and without GF. C1, C2, C4, and C5 combinations were studied for the apoptotic cell death as synergism was not observed in the C3 group with GF. 

**Figure 4:**
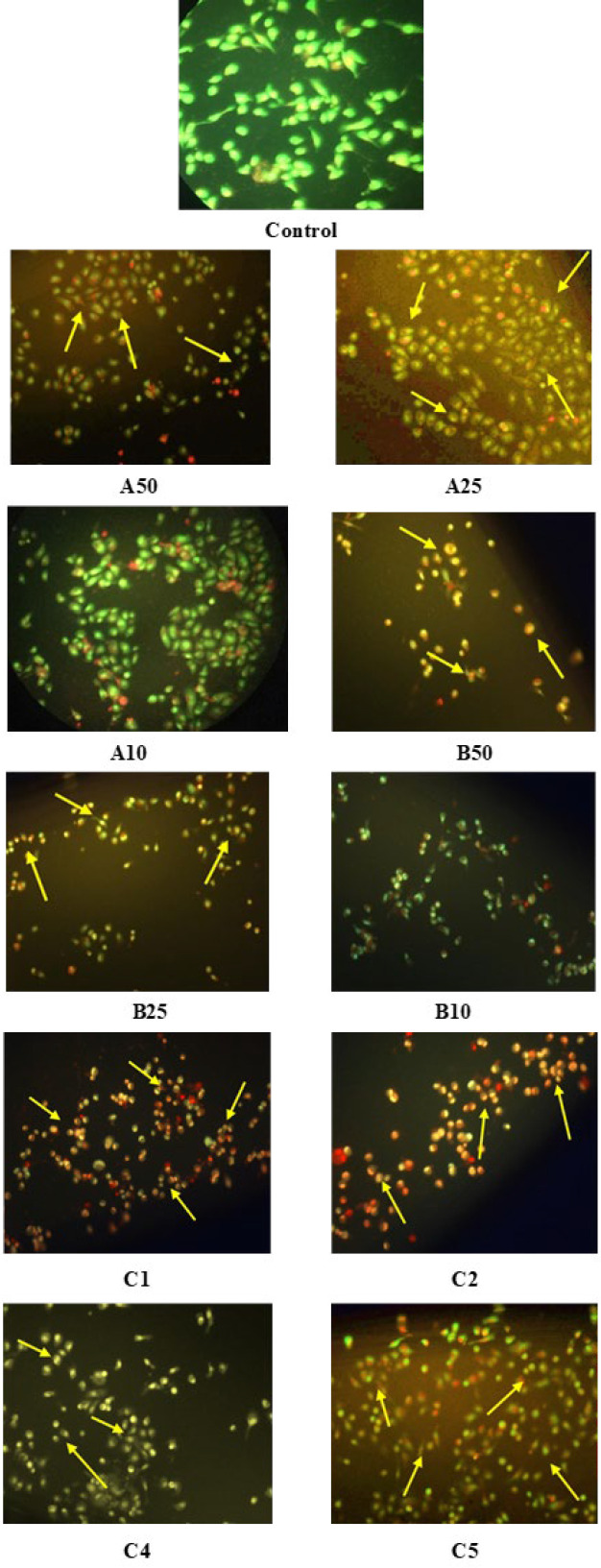
Apoptotic cell death in PC3 cells treated with Abiraterone acetate (AA) and CDRI-08 without growth factor (GF)

**Figure 5:**
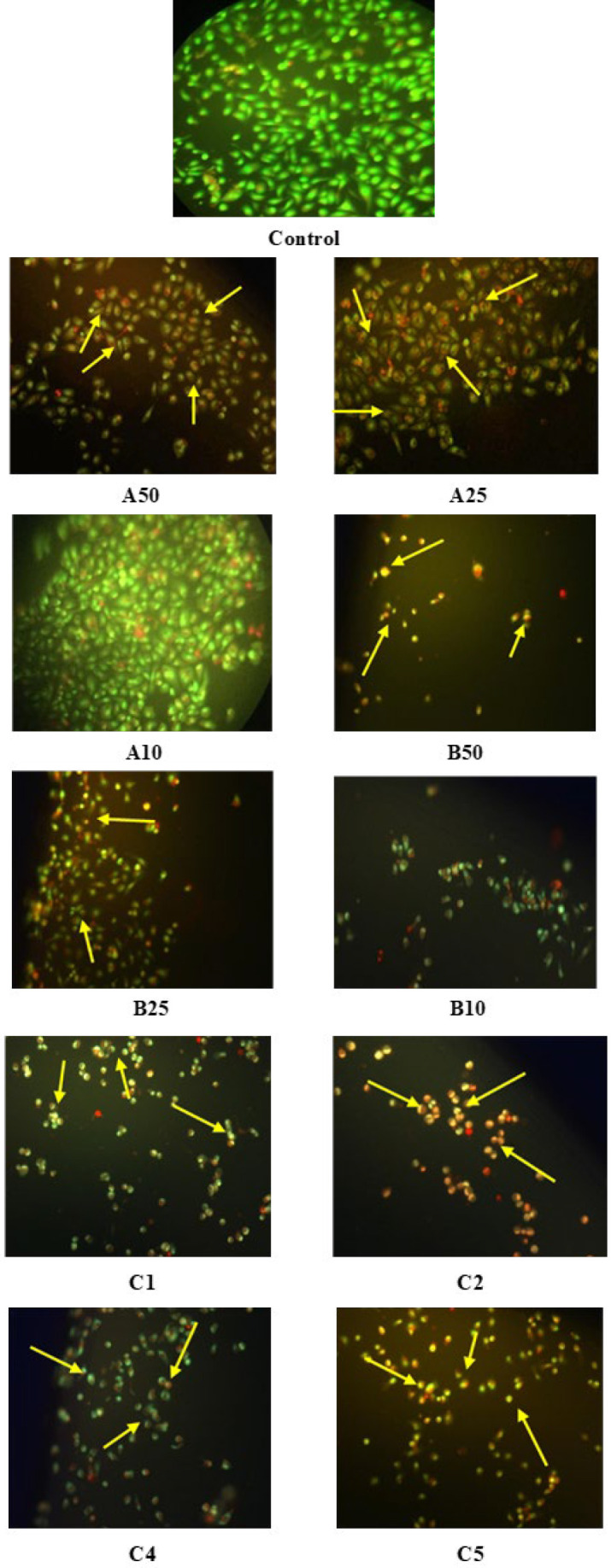
Apoptotic cell death in PC3 cells treated with Abiraterone acetate (AA) and CDRI-08 with growth factor (GF)

Observation of AO/EB stained cells under a fluorescence microscope (Figures 4 and 5) showed nuclei and chromatin condensation, with greenish-yellow and orange fluorescence indicating early and late apoptosis, respectively. In comparison, the live cells (control) showed green fluorescence. 

### Protein expression studies

**Figure 6:**
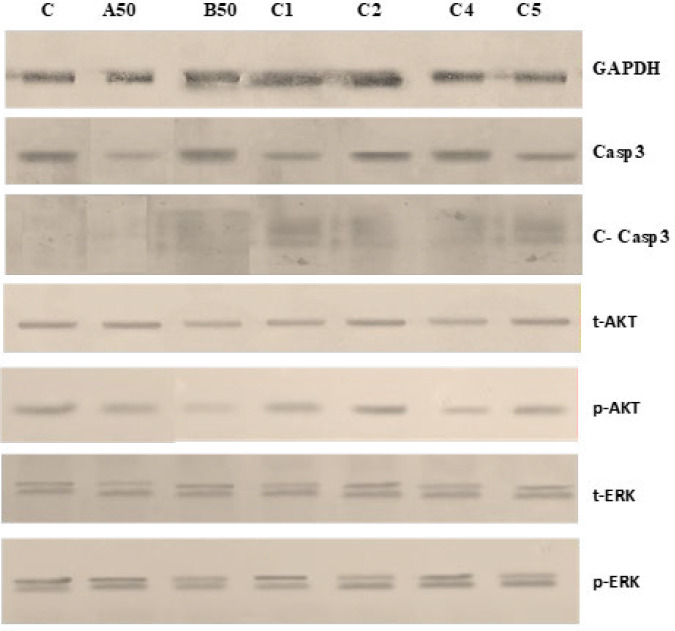
Western-blot analysis of changes in expressions of apoptotic protein and Kinase proteins

The changes in the expression of p-AKT and p-ERK and Casp3 and its active form Cleaved casp3 were studied in the individual and combination ICs treated cells with the western blot experiment (Figure 6). The C-casp3 expression level was found to be increased in CDRI-08 (IC50) individual treated cells and all the combination ICs treated cells compared to the control. The C-casp3 expression was found to be increased in the C1 and C2 combination-treated cells compared to the CDRI-08 treated cells. Thus, the study shows that the presence of CDRI-08 increased the activity of apoptotic cell death in combinations, even in the presence of GF.

CDRI-08 treated cells have decreased p-AKT expression levels, indicating an inhibitory effect on the p-AKT. The AA individual treated cells showed no inhibitory effect on the p-AKT and p-ERK. p-AKT expression was found to be reduced in C1 and C2 combination treated cells compared to other combinations treated cells. There were no changes in the expression levels of p-ERK in both the individual and the combination ICs treated cells. The AKT inhibitory effect of CDRI-08 may be an important factor for the increased cytotoxicity observed in the combination treated cells at a low dose, even in the presence of GF.

## Discussion

The PCa progresses to CRPC after ADT due to dysregulation of interlinked pathways and AR, resulting in the development of drug resistance (25). AR and androgens were found to play a crucial role even in CRPC progression and metastasis (25). The inhibition of intra-tumoral androgen synthesis targeting CYP17A1 with AA has proven to be highly effective (26). However, monotherapies have shown to cause drug resistance in CRPC patients due to dysregulation of PI3K/Akt pathway, which is one of the major clinical challenge in CRPC treatment (6). Thus, the current therapeutic research is aimed to target the complex interlinked pathways with combination therapy.

AR pathway plays a significant role in CRPC progression and many current clinical trials are aimed to discover the potential combination of ARPI with chemotherapy, Kinase inhibitors, and immunotherapy (27). Combination studies with phytocompounds have shown to sensitize the tumor cells for chemotherapy and protect the normal cells (28, 29). Although plant preparations have gained attention for various medicinal properties, screening for potential toxicity with scientific validity is attention-seeking (30). Studies with Zebrafish and its embryos have gained a lot of attention in recent years as one of the well-accepted model systems for studying genetic development, toxicity, and teratogenicity (31). In the current study, CDRI-08 has shown no toxicity and teratogenicity till 200 µg/ml in developing embryos. 

The cytotoxicity study of the AA and CDRI-08 individually against the PC3 cell line showed effective dose-dependent decrease in cell viability, with good IC50 values. The IC50 of CDRI-08, 34 µg/ml, was also found to be non-toxic as studied in the zebrafish embryos. EGF and DHT were shown to activate EGFR pathway, and AR pathway and mutations leading to drug resistance (32, 33). Both AA and CDRI-08 have shown effective cytotoxicity even in the presence of GF. The synergistic effect of AA and CDRI-08 in the presence of GF was validated with the Bliss independent model and CI. The CI value predicts the effectiveness of drug synergism (34), where the CI value equal to 1 indicates additivity, while less than 1 indicates synergism, and more than 1 indicates antagonism (35). The combination of CDRI-08 and AA showed an effective synergism with CI˂1, even in the presence of GF. The combination therapy with the kinase inhibitors, Capivasertib and Ipatasertibe, and chemotherapy, Abiraterone, Enzalutamide and Docetaxel have shown increased survival rate in mCRPC patients in clinical trials (36). The combination studies of AR inhibitors and the phytocompounds, Resveratrol, Quercetin, EGCG, Curcumin, and Genistein have shown an enhanced cytotoxic effect at lower doses with increased apoptosis and reduced chances of drug resistance (37).

Evasion of apoptosis in cancer cells was found to occur via imbalance in pro-apoptotic and anti-apoptotic proteins, deregulation in caspase activity, oxidative stress, and mutations (38). PI3K/AKT pathway was shown to increase survival in cancer cells through inactivation of proapoptotic proteins. (39). Apoptotic anticancer activity was studied with dual AO/EB staining to identify and distinguish apoptosis cells (40). In the current study, all the individual ICs treated cells showed effective apoptosis, even in the presence of GF. C1 combination group has shown a significant increase in late apoptotic cells, with and without GF. The combination ICs treated cells have shown an effective apoptotic activity compared to those treated with individual ICs of AA and CDRI-08. 

The active compound, Bacoside A, a triterpenoid (41), metabolizes into four terpenoids, Bacoside A3, Bacopaside II, Bacopasaponin C and Bacopaside X 13. In previous *in-silico* studies in our lab, the docking of bacosides with kinase receptors, EGFR, PI3K, Akt, and ERK has shown significant molecular interactions with the active site amino acids and good binding energy, indicating the inhibitory effect (42). Therefore, a protein expression study was performed to analyse the changes in the expression levels of Casp3, AKT, and ERK. CDRI-08 has shown a decrease in the expression of p-AKT both in the individual and combination ICs treated cells, in the presence of GF. The combination study with lower doses of CDRI-08 and AA showed more inhibitory effects on p-AKT expression levels. The study has shown a decrease in p-AKT expression levels, with simultaneous increase in Casp3 and C-casp3 expression levels in CDRI-08 individual and combination ICs treated cells at low doses with GF. An active PI3K/Akt pathway has been shown to decrease the apoptotic activity, resulting in cancer cell survival (43) 

A recent study with AKT inhibitors has shown inactivation of the anti-apoptotic protein BCL-2 with simultaneous activation of the pro-apoptotic protein BAD, leading to apoptosis (44, 45). Naringenin, a flavonoid from citrus, in combination with Paclitaxel was shown to increase Bax and reduce BCL-2, in PC3 cells, along with downregulation of p-ERK1/2 and p-AKT (46). Caspases are used as a marker to analyse the efficacy of cancer therapy, as they are the major mediator of apoptosis (47). 

The individual and combination ICs treated cells were not found to have an inhibitory effect on p-ERK expression levels, compared to the control. Various studies with PI3K/AKT inhibitors have been shown to indirectly activate the EGFR/ERK pathway (48). However, in the current study, AKT inhibition was not found to activate the ERK in the combination ICs treated cells. Thus, this may indicate an indirect inhibitory effect on EGFR/MAPK/ERK pathway. A recent study targeting Akt and AR pathways with AZD5363 and enzalutamide, respectively, has shown to delay the development of drug resistance with a simultaneous increase in apoptosis and cell cycle arrest (49). Thus, the current study supports the competent cytotoxic effect of CDRI-08 against androgen-independent prostate cancer cell lines, PC3, targeting the PI3K/Akt pathway, in combination with CYP17A1 inhibitor, AA, even in the presence of EGF and DHT. The synergistic effect shows the competent role of CDRI-08 and AA in combination.

The morphological changes were observed for 96 hpf under an Inverted Nikon SMZ1000 zoom microscope for uniformity.

The results were represented as mean±SD with *P<*0.05

The results were represented as mean±SD with *P<*0.05 analysed with one-way ANOVA

Drug A: AA; Drug B: CDRI-08

**Table 1:** Individual inhibitory effect of Abiraterone acetate (AA) and CDRI-08

Compounds	Inhibitory concentrations	% Inhibition	
		Without GF	With GF
CDRI-08 (µg/ml)	IC_50_ (34)	47.42	59.2
	1C_25_ (17)	28.7	27.6
	IC_10_ (7)	No effect	No effect
AA (µM)	IC_50_ (27)	48.6	47.4
	IC_25_ (13.5)	29.5	26.3
	IC_10_ (6)	No effect	No effect

The results were represented as mean±SD with *P<*0.05

**Table 2:** Combination Index (CI) of Abiraterone acetate (AA) and CDRI-08 in the presence and absence of growth factor (GF)

S.No	Combinations		Without GF	With GF
	AA (µM)	CDRI-08 (µg/ml)	CI	CI
C1	IC_25_	IC_25_	0.619	0.78
C2	IC_25_	IC_50_	0.86	0.9
C3	IC_50_	IC_10_	0.93	1.77
C4	IC_50_	IC_25_	0.85	0.86
C5	IC_10_	IC_50_	0.72	0.81

The figures represent the apoptosis-induced morphological changes in PC3 cells treated with individual ICs and combination ICs of AA and CDRI-08, compared to control, viewed under inverted phase-contrast microscope (Nikon Eclipse Ti-S, Japan) at 200 × magnification. The arrows indicate the early apoptotic cells with greenish-yellow fluorescence and late apoptotic cells with orange fluorescence

CDRI-08 ICs- A50, A25, A10; AA ICs- B50, B25, B10; C1- IC25A+IC25B, C2- IC25A+IC50B, C4- IC50A + IC25B, C5- IC10A+IC50B

The figures represent the apoptosis-induced morphological changes in PC3 cells treated with individual ICs and combination ICs of AA and CDRI-08, compared to control, viewed under Inverted Phase-- Contrast microscope (Nikon Eclipse Ti-S, Japan) at 200 × magnification. The arrows indicate the early apoptotic cells with greenish-yellow fluorescence and late apoptotic cells with orange fluorescence. The PC3 cells treated with individual and combination ICs of AA and CDRI-08 showed yellow and orange fluorescence, indicating early and late apoptosis, compared to the control. The results have shown a concentration-dependent apoptotic activity in cells treated with individual ICs of CDRI-08 and AA. The IC50 of AA and CDRI-08 treated cells have shown an effective apoptosis, which is followed by IC25 treated cells. The IC10 treated cells with 0% cytotoxic effect showed green fluorescence, indicating live cells. The C1 and C2 combination-treated cells, without GF, have shown orange fluorescence, indicating late apoptosis. The C4 and C5 combination treated cells, without GF, have shown early apoptosis with yellow fluorescence. In the presence of GF, the C1 and C4 combination-treated cells have shown early apoptosis with yellow fluorescence, while the C2 and C5 combination-treated cells have shown late apoptosis with orange fluorescence

CDRI-08 ICs- A50, A25, A10; AA ICs- B50, B25, B10; C1- IC25A+IC25B, C2- IC25A+IC50B, C4- IC50A+IC25B, C5- IC10A+IC50B

The figures represent the changes in the expression of apoptotic protein Casp3, cleaved casp3 and kinase proteins, total AKT (t-AKT), phosphorylated AKT (p-AKT), Total ERK (t-ERK), and phosphorylated ERK (p-ERK), in Control, Individual ICs, and Combination ICs treated PC3 cells

C- Control; Individual ICs A50- AA IC50, B50- CDRI-08 IC50, Combination ICs C1- IC25A+IC25B, C2- IC25A+IC50B, C4- IC50A+IC25B, C5- IC10A+IC50B

AA: Abiraterone acetate

## Conclusion

The current study shows the synergistic effect of AA and CDRI-08 at lower doses, with and without growth factors, which may reduce the chances of development of drug resistance. The study supports the drug repurposing of CDRI-08 as an effective anticancer drug for CRPC. CDRI-08, in combination with AA, an approved CRPC drug, may provide strong support for further clinical trials for combination therapy in CRPC. 

## Data Availability

No datasets were generated or analyzed during the current study.

## Declaration

We have not used any AI tools or technologies to prepare this manuscript.
